# Recreation Facility Food and Beverage Environments in Ontario, Canada: An Appeal for Policy

**DOI:** 10.3390/ijerph18158174

**Published:** 2021-08-02

**Authors:** Susan Caswell, Patti-Jean Naylor, Dana Olstad, Sara Kirk, Louise Mâsse, Kim Raine, Rhona Hanning

**Affiliations:** 1 School of Public Health Sciences, Faculty of Health, University of Waterloo, Waterloo, ON N2L 3G1, Canada; rhanning@uwaterloo.ca; 2 Institute of Applied Physical Activity and Health Research, School of Exercise Science, Physical and Health Education, University of Victoria, Victoria, BC V8W 3P1, Canada; pjnaylor@uvic.ca; 3 Department of Community Health Sciences, Cumming School of Medicine, University of Calgary, Calgary, AB T2N 4Z6, Canada; dana.olstad@ucalgary.ca; 4 Healthy Populations Institute, Dalhousie University, Halifax, NS B3H 4R2, Canada; sara.kirk@dal.ca; 5 BC Children’s Hospital Research Institute, School of Population and Public Health, University of British Columbia, Vancouver, BC V6H 3V4, Canada; lmasse@bcchr.ubc.ca; 6 School of Public Health, University of Alberta, Edmonton, AB T6G 1C9, Canada; kim.raine@ualberta.ca

**Keywords:** food environments, food policy, recreation facilities, sports clubs, nutrition guidelines, food-based guidelines

## Abstract

Canadian, municipally funded recreation/sport facilities typically have unhealthy food environments. Ontario, unlike some provinces, lacks a voluntary recreation facility nutrition policy. This study assessed the healthfulness of food environments and vending sales in 16 Ontario recreation/sport facilities and, secondarily, compared data from facilities within municipalities that banned versus permitted plastic bottled-water sales (water-ban, *n* = 8; water, *n* = 8) to test the nutritional effects of environmental policy. Concession and vending packaged food/beverage offerings and vending sales were audited twice, eighteen months apart. The products were categorized using nutrition guidelines as *Sell Most (SM)*, *Sell Sometimes (SS)*, and *Do Not Sell (DNS)*. Both water and water-ban facilities offered predominantly (>87%) *DNS* packaged food items. However, proportions of *DNS* and *SM* concession and vending beverages differed (*p* < 0.01). *DNS* beverages averaged 74% and 88% of vending offerings in water and water-ban facilities, respectively, while *SM* beverages averaged 14% and 1%, respectively. Mirroring offerings, *DNS* beverages averaged 79% and 90% of vending sales in water versus water-ban facilities. Ontario recreation/sport facilities provided unhealthy food environments; most food/beverage offerings were energy-dense and nutrient-poor. Water bans were associated with increased facility-based exposure to *DNS* beverage options. A nutrition policy is recommended to make recreation facility food/beverage environments healthier and to mitigate unintended negative consequences of bottled-water bans.

## 1. Introduction

Municipally funded recreation and sport facilities, also known in some jurisdictions as sports clubs, are an environment with a mandate to promote physical activity. Kokko [1] conceptualized recreation facilities/sports clubs as a health-promoting setting and identified nutrition-related activities as an important theme. Since healthy food environments are fundamental to supporting healthy dietary practices [2], recreation facilities are uniquely positioned to both model a healthy food environment and to promote healthy eating practices [3]. Moreover, given the high levels of child and youth attendance [4], recreation facilities, similar to schools, are an environment with the potential to support the development of healthier preferences and have a measurable, lasting influence [5,6,7].

An unfortunate paradox is the poor nutritional quality of the food prevalent in these settings, which are supposed to promote community well-being; the food environment within recreation facilities is typically unhealthy [8,9,10,11,12,13,14,15]. A key reason is the pervading availability of food vending machines and concession-style food services. Within Canada, snack vending machines typically contain packaged, non-perishable snack foods low in nutrients and high in fat, sugar, salt, and calories, such as candy, chocolate bars, and chips/crisps, while beverage vending machines typically contain sugar sweetened carbonated beverages, sports drinks, energy drinks, bottled water, and artificially sweetened ‘diet’ beverages, usually in plastic bottles greater than 500 mL. In addition, concessions offer predominantly ultra-processed [16], low-cost prepared foods, such as French fries, and limited, if any, healthier options.

Places where children spend time, learn, and play should support healthy choices and offer consistent messaging [17,18]. In Ontario, the school food and beverage policy (P/PM 150) mandates that the foods offered for sale meet nutrition guidelines [19]; however, recreation facilities have no such policy. Consistency across these environments would allow the messages to be internalized and to help normalize healthier food choices [5]. Furthermore, given that these facilities are government funded, it is a reasonable expectation that they reinforce broader population health promotion and do not provide environments that conflict with public health goals [1].

There is increasing recognition by health professionals and policymakers that recreation facilities have the potential to support healthy eating [9,10,20,21,22]. As a result, there has been a move to implement nutrition policy within recreation and sport facilities in Canada, with a number of provinces implementing voluntary nutrition policies or guidelines [23,24,25]. Ontario, with some municipal-level exceptions, has yet to implement recreation facility nutrition policies or guidelines. Voluntary nutrition guidelines have been associated with a shift towards healthier food environments, especially where there were capacity building strategies to support policy implementation [26,27,28]. However, even where there was a positive shift in the healthfulness of food environments, most foods offered fell well below the recommendations [26,27,28].

As Allender et al. [29] found in Victoria, Australia, promoting healthy food may not be a priority for local governments; key informants reported that food policies lacked relevance or was outside of their mandate, a sentiment also reflected elsewhere [6,28,30,31]. There is also an increasing body of evidence on perceived barriers to policy development and implementation within recreation facilities, including competing priorities, fears of financial losses, lack of local champions, poor stakeholder buy-in, and cultural norms that favour unhealthy foods [15,28,29,32,33]. Ontario can learn from the experience of others.

Data for the current study were collected as part of the larger cross-Canada nutrition policy intervention trial Eat, Play, Live (EPL)*,* described elsewhere [26,27]. The province of Ontario served as the comparative province for EPL, being without specific nutrition guidelines or policies governing municipal recreation and sport facilities. Most studies on food environments, with a few exceptions [15,29,30], examine what is offered for sale, but EPL also looked at what was actually sold. The primary objective of this paper was to provide a snapshot of the healthfulness of the food environment in a sample of municipally funded recreation and sport facilities across the province of Ontario, Canada, and how that was reflected in vending sales.

In Ontario, municipal concerns about the environmental impact of single-use plastic bottles have led several jurisdictions to institute bans on the sale of bottled water within municipally funded buildings. Outside the control or intention of the investigators, half of the EPL Ontario facilities were within these jurisdictions. This provided a natural experiment to examine whether the bottled water ban policy affected the healthfulness of the facility food and beverage environment and beverage sales. Therefore, the purpose of this study is two-fold, and the secondary objective was to compare the nutrition environments and vending beverage sales of the cohort of participating facilities within municipalities with a bottled-water ban policy versus the cohort of facilities without such a policy. We hypothesized that, since the water-bottle ban policies did not limit the sale of other beverages in plastic bottles, the beverage environment would be less healthy in the water-ban policy cohort while there would be no difference in the healthfulness of the food offered.

## 2. Materials and Methods

### 2.1. Study Design

This project was guided by a larger research group and a smaller provincial advisory group comprised of health professionals.

Cross-sectional descriptive data on the food environments in municipal recreation and sport facilities in Ontario were collected on two occasions, corresponding to the baseline and follow-up times of the intervention provinces in the larger EPL trial. Facilities were eligible to participate in EPL if they had a concession that was open year-round, had neither taken action to change their facility food environment nor participated in studies targeting change in recreational food environments in the previous five years, and had no plans to change food and beverage services for the eighteen-month duration of the study. Clinical Trials Registration: ISRCTN14669997 3 July 2018 (retrospectively registered).

### 2.2. Recruitment

The provincial study partner, Parks and Recreation Ontario, distributed an email containing a participation invitation letter to senior municipal recreation contacts across the province. The invitation was forwarded to recreation and sport facilities believed to be eligible to participate. After this, the EPL research team followed up with the facility contacts by telephone. Of the fifty-three sites approached, seventeen were recruited to participate. Of the non-participating facilities, five did not respond to contact attempts; two declined to participate, citing poor timing and concession restructuring; one did not submit a participation agreement; and twenty-eight did not qualify. Hence, 68% of eligible facilities participated.

### 2.3. Data Collection and Measures

Primary outcomes were measured in Ontario between February and April 2016 and between September 2017 and January 2018. The measures included vending product profiles, concession packaged product and menu profiles, concession food environment assessment, and vending sales. A requirement of the audit was that concessions be open to patrons. With concession hours closely linked to facility traffic volume, audit times varied according to facility (i.e., some facilities only had concessions open evenings and weekends). Audit data were collected by the primary author (S.C.) and included both vending and concession food and beverages, as detailed below.

#### 2.3.1. Vending

Four vending machines per facility were randomly selected using a remotely generated computer randomization sequence: two beverages and two snacks. A detailed description of the packaged food and beverage products available for sale, including brand, product variety/type, size, flavour, and price was recorded. The healthfulness of products was then assessed by classifying each one according to established British Columbia, Canada Ministry of Health Guidelines as *Sell Most*, *Sell Sometimes*, and *Do Not Sell* [24]. *Sell Most* are the healthiest options, high in essential nutrients and low in sodium, sugar, and fat. *Sell Sometimes* products provide essential nutrients but with higher amounts of sodium, sugar, and/or fat. *Do Not Sell* have high fat, sodium, or sugar and are nutrient poor [24]. Test-retest and inter-rater reliability for this audit process was previously shown to be high (≥0.88) [11].

#### 2.3.2. Concession

Packaged food and beverage items for sale in concessions were recorded and evaluated using the same categorization used for vending machines. Products available at point-of-purchase, defined as within one metre of a cash register, were also documented and assessed separately.

Concession food environments were measured using a validated adapted version of the Nutrition Environment Measures Survey-Restaurant reduced item audit (rNEMS-R) Fast Casual and Fast Food Summary [34]. Food environment scores using rNEMS-R can range from −4 to +48 for the Fast Casual Summary and from −10 to +51 for the Fast Food Summary, with higher scores indicating greater availability and lower cost of healthy options, and support making healthier choices [34]. Main dish items were defined as items with a protein source and at least one other food group. As a menu nutritional analysis of prepared items was not feasible in this context, a harmonized food and beverage classification scheme was created to provide a consistent basis to classify the healthfulness of these main dish items. Healthy main dish options were given a score of three, and lower scores indicated less healthfulness. Full details of the classification scheme are presented elsewhere [26]. In addition, to allow for greater differentiation between facilities, the availability of specific marker foods (e.g., fresh fruit and vegetables, low-fat milk, and fried and baked French fries) and preparation equipment were recorded.

#### 2.3.3. Vending Sales

Snack and beverage vendors provided itemized sales data for products sold in vending machines, targeting a two-week period around concession and vending audits. The time frame ranged from fourteen to thirty-one days due to machine refill schedules and was normalized to two weeks.

### 2.4. Analyses

As Ontario was the non-intervention control province for EPL, no differences in audit and sales data were observed between baseline and follow-up data collection [27]. Hence, the data collected at both time points were averaged for the current analyses. Descriptive and comparative statistics were calculated using IBM SPSS Statistics for Windows 26 and Microsoft Excel. A *t*-test, assuming unequal variance, was used to determine the differences between facilities within the bottled water ban cohort (H_2_O Ban) and facilities without a bottled-water ban (H_2_O) for their mean proportion of food/beverage items offered or sold by category (*Sell Most*, *Sell Sometimes*, and *Do Not Sell*) for each of vending, concession packaged, or point-of-purchase. Items with no nutritional value (e.g., tea, coffee, gum, and throat lozenges) were excluded.

### 2.5. Ethics Clearance and Consent

This study received ethics clearance from the University of Waterloo Office of Research Ethics (ORE file #20913) as well as the research ethics boards at the University of Victoria, the University of British Columbia, the University of Alberta, and Dalhousie University. Facility representatives provided written, informed consent for facility participation.

## 3. Results

### 3.1. Participating Facilities

Between baseline and follow-up, one facility closed its concession, making it ineligible. Therefore, sixteen Ontario facilities were included of the seventeen recruited (See Table 1 for the facility characteristics). Eight facilities were located in municipalities with a regulated policy banning the sale of bottled water (H_2_O Ban), and eight were in municipalities with no restrictions (H_2_O). All sixteen facilities provided beverage vending machines ($\;\bar{x}\;$ = 3.5; $\;\tilde{x}\;$ = 3; range 1–7 per facility), while twelve also provided snack vending (of these, $\;\bar{x}\;$ = 2; $\;\tilde{x}\;$ = 2; range 1–4). There were no changes to the number of vending machines between baseline and follow-up, with the exception of one facility adding an ice cream vending machine. Participating facilities had concessions operating twelve months of the year: fifteen facilities with a single concession offering both packaged and prepared foods and one facility with a second concession offered packaged snacks and beverages but not prepared menu items.

### 3.2. Site Audits

Table 2 provides a breakdown for the H_2_O and H_2_O Ban cohorts for vending and concession packaged food and beverages, and concession point-of-purchase items. All packaged food and beverage offerings were profiled and classified based on existing provincial guidelines as *Sell Most*, *Sell Sometimes*, and *Do Not Sell* [24]. It should be noted that bottled water is categorized as *Sell Most*.

### 3.3. Vending Offerings

For beverage vending, there was a significant difference between the H_2_O and H_2_O Ban cohorts for mean proportions in both the *Sell Most* and *Do Not Sell* categories that were offered (See Table 2). H_2_O Ban facilities had a higher mean proportion of *Do Not Sell* and a lower mean proportion of *Sell Most*. However, even in the H_2_O cohort facilities, *Do Not Sell* beverages still accounted for over 70% of the vending beverages offered.

For snack vending, product profiling categorized most products offered as *Do Not Sell* for both cohorts (See Table 2). Almost none of the product offerings were *Sell Most*, and only a small proportion were *Sell Sometimes*. There were significant differences between the cohorts for vending snack categories, with the H_2_O Ban facilities having proportionately more *Sell Sometimes* and fewer *Do Not Sell* items.

### 3.4. Concession Packaged Food, Beverages, and Point-of-Purchase Offerings

The mean number of packaged beverage options for H_2_O and H_2_O Ban facilities was 19.9 (SD = 5; $\;\tilde{x}\;$ = 20; range 10–28) and 21.6 (SD = 6.1; $\;\tilde{x}\;$ = 21; range 15–34), respectively. As with vending beverages, there was a significant difference between the H_2_O and H_2_O Ban cohorts for mean proportions of beverages within both the *Sell Most* and *Do Not Sell* categories (See Table 2). Again, H_2_O Ban facilities had a higher mean proportion of *Do Not Sell* and a lower mean proportion of *Sell Most items*.

The mean number of concession packaged snack options for H_2_O and H_2_O Ban facilities was 24.3 (SD = 3.7; $\tilde{x}$ = 24; range 20–31) and 24.4 (SD = 13.8; $\;\tilde{x}\;$ = 23; range 6–48), respectively. For concession packaged food, there were no significant differences in healthfulness categorization between the H_2_O and H_2_O Ban cohorts; both offered predominantly *Do No Sell* items. As seen in Table 2, only about 1% of concession packaged food were *Sell Most* foods in both cohorts.

Most (*n* = 13) concessions offered packaged point-of-purchase snack and beverage items at the register. The mean numbers of point-of-purchase snack and beverage items for H_2_O (*n* = 8) and H_2_O Ban (*n* = *5*) facilities were 14.8 (SD = 12.8; $\;\tilde{x}\;$ = 11; range 2–40) and 10.4 (SD = 12.3; $\;\tilde{x}\;$ = 5; range 0–33), respectively. There was no significant difference in the number of items offered between the two cohorts (*p* = 0.942). There were also no significant differences in categorization between point-of-purchase snack and beverage offerings and between the H_2_O and H_2_O Ban cohorts, with both offering predominantly *Do Not Sell* items (See Table 2).

### 3.5. Concession Menus and Food Environment

Table 3 summarizes the menu marker foods and entrée scoring. Six H_2_O and five H_2_O Ban facilities used deep fat fryers and offered deep fried menu options, including French fries, onion rings, and chicken fingers. No facilities in either cohort offered baked French fries, baked chips, or healthy main dish salads. Only one facility offered salad as a side dish, and low-fat dressing was not an available option. All facilities in both cohorts offered 100% fruit juice, milk (not low-fat), and chocolate milk as beverage options. Fruit was available at two H_2_O facilities; however, it was listed as a menu item at only one of those facilities. Fresh vegetables (e.g., veggie sticks) were a menu option at one H_2_O facility; however, none were available for purchase at either audit timepoint.

**Figure 1:**
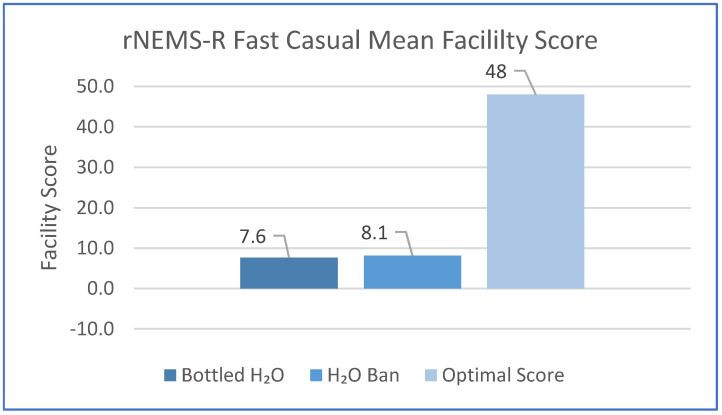
rNEMS−R Fast Casual mean facility score according to H_2_O and H_2_O Ban.

**Figure 2:**
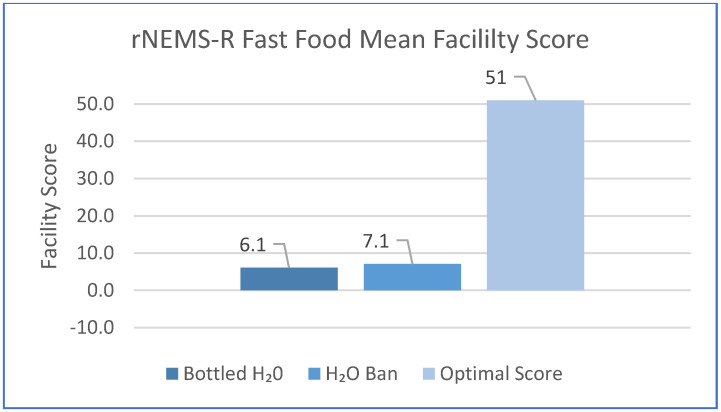
rNEMS−R Fast Food mean facility score according to H_2_O and H_2_O Ban.

The mean facility Fast Casual and Fast Food summary scores are shown in Figure 1 and Figure 2, respectively. There were no significant differences between H_2_O Ban and H_2_O facility scores. Importantly, the scores indicate a poor nutrition environment for both cohorts.

### 3.6. Vending Sales

Beverage vending sales reflected the profiles of products offered for sale when analyzed by both mean proportion of items sold for each category and by mean proportion of sales dollars for each category. Significant differences between the H_2_O and H_2_O Ban facilities can be seen for both *Sell Most* and *Do Not Sell* (See Table 4). The mean proportion of *Sell Most* items sold and the mean proportion *Sell Most* sales dollars were higher in H_2_O facilities, whereas the mean proportion of *Do Not Sell* items sold and the mean proportion *Do Not Sell* sales dollars were higher in H_2_O Ban facilities. Even in H_2_O facilities, almost 80% of items sold and sales dollars came from *Do No Sell* beverages.

When considering vending snack sales, the proportion of items sold was also significantly different between the H_2_O Ban and H_2_O facilities in the *Do Not Sell* and *Sell Sometimes* categories, reflecting the slight but significant difference in H_2_O facility offerings above (See Table 4). However, within each cohort, there was an apparently higher mean proportion of *Do Not Sell* items sold than the mean proportion of *Do Not Sell* items offered for sale.

## 4. Discussion

Recreation and sport facilities are an important setting when considering influences on eating practices. As an environment in which many children spend considerable time, unhealthy food options have the potential to do harm [37]. The results clearly demonstrate that the food environment is unhealthy in these facilities. The majority of the items sold were ultra-processed, energy-dense, and nutrient-poor, and it is noteworthy that vending sales data indicated that the unhealthy food environment within the participating facilities translated to food purchases. Our data also demonstrated the power of policy. Not only was the healthfulness of the food offered and sold in Ontario worse than in other provinces with nutrition policy [26] but also a municipal policy to ban water in plastic bottles while allowing other drinks in plastic bottles was evidenced in the adverse effect it had on the food nutrition profile in the H_2_O Ban facilities.

### 4.1. Facility Food Environment

The primary objective of this research was to describe the food environment in Ontario’s municipally funded recreation and sport facilities. Packaged snack and beverage offerings through vending machines and within concessions were predominantly categorized as *Do Not Sell*. Food environment scores were well below the ideal for both rNEMS-R Fast Casual and Fast Food, indicating that few healthy options were offered in concession food services. None of the facilities offered prepared menu items that received a healthy score. Marker foods indicated a predominance of unhealthy choices and limited, if any, availability of healthier options. Unfortunately, this picture is not atypical of recreation and sport facility food environments across Canada [10,12,26,28] or globally [13,14,38]. It is also relevant that including healthy items on the menu boards did not translate into actual availability; the absence of the menu item veggie sticks, noted during both audits of one facility, was described by the staff as the norm rather than the exception.

The sales data for vending snacks and beverages were also predominantly from the *Do Not Sell* category, supporting prior research suggesting that food and beverage purchases reflect their proportionate availability [39,40]. If most items are unhealthy, the probability of purchasing a healthy option may be lower. In light of current findings that product availability reflects product sales, improving the healthfulness of recreation food environments should be a priority.

### 4.2. H_2_O vs. H_2_O Ban

The presence of a single-use plastic bottled-water ban in half of the participating facilities presented the opportunity to assess unintended consequences of environmental sustainability policy with health promotion implications. Therefore, a second objective of this study was to examine the associations between municipal bottled-water bans and the healthfulness of recreation and sport facility food and beverage availability and sales. One of the most interesting and novel findings was that, while overall both the H_2_O and H_2_O Ban facilities were clearly unhealthy food environments, there were significant differences between the two cohorts. Where plastic water bottles were banned, empty vending slots and concession beverage options were replaced with less healthy beverages also in plastic bottles. While the environmental and practical intentions of plastic water bottle bans is to divert plastic from landfills, it is clear from this data that unintended impacts on food environments and health may not have been fully considered [41].

As would be expected, the restriction of bottled water (a healthy beverage choice), meant that the preponderance of beverages offered and sold through vending machines and concessions were less healthy options. As water was categorized as *Sell Most*, the restriction on the sale of bottled water resulted in the facilities with a ban having a higher proportion of *Do Not Sell* beverages and a lower proportion of *Sell Most* beverages, both available and sold. The one H_2_O Ban facility offering *Sell Most* beverages was able to do so as they sold carbonated water in glass bottles, so it did not contravene the plastic bottle restriction. It is also important to note that all participating facilities had water fountains for refillable bottles, so while the availability of bottled water was not a limiting factor for drinking water, it required pre-planning for patrons. Anyone wanting to purchase a beverage would have few healthy options.

How differences in product offerings translated to sales is of particular importance. It was observed that the proportion of *Do Not Sell* and *Sell Sometimes* vending snack items sold aligned with the proportions of products offered in both cohorts. This suggests that sales mirrored product availability in both cohorts, a pattern demonstrated elsewhere [39,40]. This pattern was also true of vending beverages sales. Hence, in the absence of water, there was not a proportional shift in sales to the next healthiest *Sell Sometimes* choice but rather sales reflected the dominant *Do Not Sell* beverage availability. The variation in facilities’ sizes and services and the lack of pre-policy data does not allow us to calculate the absolute differences in sales. At the very least, the H2O Ban increased exposure to unhealthy *Do Not Sell* beverages and all but eliminated *Sell Most* vending and concession beverage options for facility patrons. Paradoxically, the bottled water ban may encourage the purchase of alternate beverages, also packaged in single-use plastic bottles, thereby increasing consumption of sugar-sweetened beverages. With newer environmentally sustainable packaging options, there may be ways forward where both goals can be met [42,43]. For example, bio-based bottles would allow for the sale of bottled water while supporting environmental goals [44].

### 4.3. A Call for Nutrition Policy

This study provides an example of how recreation and sport facilities operate in the absence of nutrition policy. The unhealthy food environment revealed in this study points to the necessity and value of policy action to provide an environment that encourages both the development and reinforcement of healthy eating practices among children and youth. Voluntary guidelines are insufficient. Voluntary policy is an acknowledgement of the value of changing the food environment without the leverage of accountability and monitoring. Other Canadian provinces have instituted a voluntary nutrition policy for recreation and sport facilities in combination with capacity building and nudging approaches to encourage healthier choices [8,26,27,28,39,45,46]. However, while they demonstrated success in shifting the healthfulness of food offerings, the magnitude of change was small and food environments remained largely unhealthy [26,28,46]. The positive findings with low-intensity interventions have to be examined in the face of the overwhelmingly unhealthy food environments that persist. To see the level of change that would support healthier eating patterns and to have an impact on children’s food practices, policy approaches with more accountability appear to be warranted [5,22,47,48].

One of the primary barriers to healthier food environments in recreation and sport settings is the entrenched cultural norms attached to the expected food experience within these facilities [15,38,49]. Changing the food environment requires a paradigm shift. While there is no doubt that a multi-faceted approach is needed, an important tool to combat these norms is a mandated policy [18,22,50,51]. Policy can play a powerful role in supporting environments that encourage learning healthy preferences early in life [48]. Lessons learned from earlier research indicate that policies can both support the development of healthier food preferences and alter norms in favour of a healthier eating environment [52]. Hawkes and colleagues’ [48] theory of change identifies key nutrition policy mechanisms and indicates that environments are central mediators between preferences and behaviour; two of the policy mechanisms they identified are particularly relevant to the recreation and sport environment. The first mechanism is to provide environments that support food, social, and information for healthy-preference learning [48]. The second is to influence availability and presentation to encourage reconsideration of existing unhealthy preferences when making food choices, particularly at point-of-purchase [48]. Although preferences can be changed over time, it is easier to influence behaviour initially than to change it once established [53].

So what options are available for Ontario? There is clearly a desire amongst the Ontario public health community to improve the healthfulness of the recreation and sport facility food environment and to support positive change. This is evidenced by programs such as The Healthy Kids Community Challenge, the publication of the Nutrition Resource Centre’s ‘Getting Started with Healthy Eating in Your Recreation Centre’, and the Ontario Food and Nutrition Strategy [21,54,55]. All three highlight the importance of encouraging healthy eating practices by ensuring supportive environments. However, while Heathy Kids Community Challenge initiatives, such as posters, were picked up in some participating facilities, the impact on offerings and sales was negligible.

Restricting the food and beverages sold based upon nutrient content is one policy option. This approach has been taken in schools, and evidence indicates that it successfully influences eating practices in children and adolescents [47,56,57,58,59]. Fung et al. [47] indicated that school nutrition policy provided “real world” evidence at a population level for the value of mandated policies promoting heathy eating. The Food-EPI Canada study, which used the Food Environment Policy Index tool to examine food environment policy in Canada, recommends extending Ontario’s policy approach with mandatory harmonized guidelines across schools, childcare settings, and recreation settings as a prioritized provincial-level action affirming the value of a legislative approach for this environment [22].

There are regions within Ontario that have implemented nutrition policy initiatives to support a healthier eating environment within local recreation and sport facilities. Additionally, there are regions that have removed concessions, although vending machines with unhealthy options remain, so this is only a partial response. Mandated provincial policy would ensure supportive environments across Ontario, not just in those regions with the political will to enact nutrition policy. Often, the children and youth participating in sports travel to other regions and experience different recreation and sport facility food environments. The lack of an overarching provincial or federal government level approach results in regional inequities and inconsistent messaging around healthy eating across municipally funded community food environments throughout the province [17,29,30]. Furthermore, in Ontario, as in other jurisdictions such as Australia, a localized recreation and sport facility nutrition policy is uncommon [32]. Patrons of those Ontario communities that are not proactive or without champions to encourage and support the inclusion of healthy options within the municipal recreation and sport facility food environment are vulnerable to vendors and facility management that do not prioritize nutrition, making it is highly unlikely that efforts will be successful [60].

Our results demonstrated that an enforced, regulated policy does indeed have an impact. This was seen with the policy banning single-use plastics for water but not banning single-use plastic sugar-sweetened beverages. The healthfulness of the nutrition environment shifted, as did the healthfulness of patron beverage purchasing. Nutrition policy at the provincial or national levels with appropriate implementation, capacity-building, and enforcement supports could mitigate such conflicts with local agendas [50]. Without such overarching policy, healthy recreation food environments may be hindered by local political agendas, limited resources, regional public health priorities, community pressures, concession vendors, vending suppliers, and the food and beverage industry [5,6,29] and continue to result in recreation environments that support unhealthy eating behaviours. However, if, for example, a provincial policy required healthful beverages and banned single-use plastic bottles, this could prompt the use of another form of packaging for water.

One final yet critical consideration is that the lack of nutrition policy also leaves the determination of what is considered a “healthy” choice open to interpretation from vendors, facility managers, and patrons [6]. During on-site audits, vendors shared a sincere belief that many of the menu options provided were indeed “healthy”, sadly, a belief that was not supported by the evidence. In previous work, the authors heard from adolescent hockey players exposed to the recreation facility food environment that marketing and coaching recommendations gave some foods a “health halo”, further supporting the necessity of objective nutrition classification of the food and beverages within this environment [52].

## 5. Strengths and Limitations

This study is the most comprehensive description of the recreation and sport facility food environment in Ontario—the most populous and largest province in Canada—to date. It provides an example of this environment in the absence of policy to provide a comparator from environments with policies (as per Olstad et al. [27]) and as a benchmark for anticipated change. This research is also the first to assess recreation and sport facility food environments across Ontario and the association of single-use plastic water bottle restrictions with the quality of the food/beverage environment. The critique of the siloed approach to environmental policy in the absence of public health considerations gives concrete support for coordinated policy. The study was restricted to volunteer facilities and excluded facilities that had municipal-level nutrition policies in place or plans to implement such a policy for the duration of the study. Nevertheless, we did recruit almost two-thirds of eligible facilities, e.g., those with food concessions operating year-round, though we recognize that this excluded facilities that offered only seasonal concessions. Thus, while the sixteen participating facilities may not be representative of facilities across Ontario, regions across the province were represented, suggesting that unhealthy recreation and sport facility food environments are indeed a provincial issue. Facilities self-selected and, therefore, likely differed in ways that could not be accounted for, other than municipal bottled water policy. Furthermore, there were limitations in the audit process. For example, staff were not blinded to the study design; thus, they may have been aware of the data collection dates, and adjusted offerings. Indeed, one staff member did indicate that this happened. Nevertheless, the predominantly unhealthy offerings suggest that adding or substituting healthy foods was uncommon.

## 6. Conclusions

In an environment centred on healthy, active living, the prevailing unhealthy food environments in Ontario’s municipally funded recreation and sport facilities must be addressed. In the absence of nutrition policy, most food and beverage offerings were energy-dense and nutrient-poor, a finding mirrored in vending packaged food and beverage sales. Moreover, the healthfulness of the food environment was found to be vulnerable to unintended consequences of municipal-level policy banning (single-use, plastic) bottled water, which, ironically, also demonstrated the power of mandated policy. Reducing the availability of convenient, portable water negatively shifted the healthfulness of beverage offerings within facilities in jurisdictions regulating water in single-use, plastic bottles. A mandated nutrition policy approach is recommended at the provincial level to align with public health guidance, to shift the nutrition healthfulness positively, and to support the overall health of those enjoying recreation and sport facilities.

## Figures and Tables

**Table 1:** Facility characteristics according to H_2_O and H_2_O Ban policy conditions.

Characteristic	H_2_O	H_2_O Ban
Facilities (*n*)	8	8
Community size ^a^ (% of facilities)		
Rural	0.0	0.0
Small population centre	12.5	12.5
Medium population centre	37.5	25.0
Large urban population centre	50.0	62.5
Facility size ^b^ (% of facilities)		
Small	0.0	12.5
Medium	37.5	25.0
Large	62.5	62.5
Vending machines (mean number)		
Vending machines	5.9 (range 2–15; $\tilde{x}$= 5)	6.6 (range 2–11; $\tilde{x}$= 7)
Beverage vending machines	3.9 (range 1–7; $\tilde{x}$= 4)	3.8 (range 1–6; $\tilde{x}$= 4.5)

^a^ Classified according to Statistics Canada values and definitions [35,36]. ^b^ A small facility had at least one minor or major amenity; a medium facility had at least two amenities, one of which was major; and a large facility had at least three amenities, two of which were major. Major amenities: ice rink, swimming pool, multiple courts (e.g., tennis, squash/racquetball), and theatre (e.g., movie, performing arts). Minor amenities: community and multi-purpose room, fitness room, and small specialty area such as a small climbing wall or gymnastics room.

**Table 2:** Packaged food and beverages: mean proportion of offerings according to H_2_O and H_2_O Ban policy conditions.

	H_2_O Mean % (SD)	H_2_O Ban Mean % (SD)	*p* Value ^a^
Concession packaged snack foods			
Facilities (*n*)	8	8	
Sell Most	0.7 (1.3)	1.1 (2.2)	0.684
Sell Sometimes	5.2 (6.8)	5.0 (3.2)	0.813
Do Not Sell	94.1 (7.5)	94.3 (3.7)	0.932
Concession point-of-purchase food & beverages			
Facilities (*n*)	8	5	
Sell Most	3.4 (5.5)	0.7 (1.5)	0.218
Sell Sometimes	9.7 (11.8)	7.0 (5.4)	0.579
Do Not Sell	86.9 (15.2)	92.4 (5.3)	0.375
Concession packaged beverages			
Facilities (*n*)	8	8	
Sell Most	7.3 (1.9)	0.6 (1.2)	<0.001 *
Sell Sometimes	16.0 (7.0)	12.5 (5.5)	0.289
Do Not Sell	76.7 (7.0)	86.9 (5.3)	0.006 *
Vending packaged snack foods			
Facilities (*n*)	5	7	
Sell Most	0.7 (0.8)	4.3 (4.3)	0.070
Sell Sometimes	3.1 (2.0)	8.6 (3.9)	0.012 *
Do Not Sell	96.3 (1.3)	87.1 (6.9)	0.011 *
Vending beverages			
Facilities (*n*)	8	8	
Sell Most	13.5 (8.1)	0.6 (1.8)	0.002 *
Sell Sometimes	12.7 (6.6)	11.4 (4.9)	0.664
Do Not Sell	73.8 (11.8)	88.0 (4.6)	0.011 *

* *p* < 0.05.; ^a^ *t*-test comparing facility means.

**Table 3:** Concession menu offerings ^a^ according to H_2_O and H_2_O Ban policy conditions

	H_2_O	H_2_O Ban
Facilities (*n*)	8	8
Marker foods (% of facilities offering each of the marker foods & beverages)		
Low fat white milk	0	0
White milk (2%, whole)	100	100
Chocolate milk	100	100
100% Juice	100	100
Fried French fries	75	62.5
Baked French fries	0	0
Potato chips—Regular	100	100
Potato chips—Baked	0	0
Fresh fruit	25.	0
Fresh vegetables	12.5	0
High fat side dishes	87.5	62.5
Healthy main dish salad	0	0
Prepared entrées		
Mean # of Entrées (SD; Median; range)	6.7 (5.8; 3.5; 1–16)	3.5 (4.9; 2.5; 1–15)
Entrée healthfulness score (# of facilities)		
Score = 3	0	0
Score = 2	0	0
Score = 1	37.5	12.5
Score = 0	100	75
Score < 0	62.5	37.5

Scoring: Maximum 3 (healthy)–<1 (least healthy); ^a^ for ease of presentation, the data shown are for Time 2 data collection only.

**Table 4:** Vending sales according to H_2_O and H_2_O Ban policy conditions.

	H_2_O Mean % (SD)	H_2_O Ban Mean % (SD)	*p* Value ^a^
Snack vending sales			
Proportion of Items Sold			
Facilities (*n*)	3	5	
Sell Most	0.2 (0.3)	3.4 (3.0)	0.076
Sell Sometimes	1.2 (1.0)	4.9 (2.1)	0.028 *
Do Not Sell	99.3 (0.4)	92.6 (2.0)	0.002 *
Beverage vending sales			
Proportion of Items Sold			
Facilities (*n*)	7	5	
Sell Most	14.8 (2.3)	0 (0.0)	<0.001 *
Sell Sometimes	6.4 (5.1)	9.3 (4.0)	0.322
Do Not Sell	78.8 (5.0)	90.4 (4.6)	0.002 *
Proportion of Sales Dollars			
Facilities (*n*)	7	5	
Sell Most	13.5 (2.5)	0 (0.0)	<0.001 *
Sell Sometimes	6.3 (5.1)	8.9 (3.7)	0.353
Do Not Sell	80.3 (4.4)	91.1 (3.7)	0.001 *

* *p* < 0.05; ^a^ *t*-test comparing facility means.

## Data Availability

The data presented here are available from the corresponding author upon reasonable request.
